# Left Ventricular Myocardial Function in Hemodialysis and Nondialysis Uremia Patients: A Three-Dimensional Speckle-Tracking Echocardiography Study

**DOI:** 10.1371/journal.pone.0100265

**Published:** 2014-06-24

**Authors:** Ran Chen, Xia Wu, Li-Jun Shen, Bei Wang, Ming-Ming Ma, Yuan Yang, Bo-Wen Zhao

**Affiliations:** 1 Department of Diagnostic Ultrasound and Echocardiography, Sir Run Run Shaw Hospital, Zhejiang University College of Medicine and Sir Run Run Shaw Institute of Clinical Medicine of Zhejiang University, Hangzhou, China; 2 Department of Radiology, Sir Run Run Shaw Hospital, Zhejiang University College of Medicine and Sir Run Run Shaw Institute of Clinical Medicine of Zhejiang University, Hangzhou, China; 3 Department of Ultrasound, The First People's Hospital, Hangzhou Xiao Shan District, Hangzhou, China; Temple University, United States of America

## Abstract

**Background:**

Several studies have demonstrated that uremic patients who have preserved left ventricular ejection fraction (LVEF) could still have the potential for systolic dysfunction. The aim of this study was to assess the differences between the left ventricular (LV) myocardial function in hemodialysis and nondialysis uremic patients based on three-dimensional speckle-tracking echocardiography.

**Methods:**

The study population consisted of 35 maintenance hemodialysis patients (the hemodialysis group), 30 uremic patients who were hospitalized for the creation of a primary arteriovenous fistula (the nondialysis group), and 32 healthy volunteers. All of the patients had normal left ventricular ejection fractions (i.e., 55% or greater). Three-dimensional speckle tracking echocardiography was performed to assess the left ventricle's global three-dimensional strain, regional longitudinal strain, circumferential strain, and radial strain.

**Results:**

The left ventricular regional longitudinal strain, radial strain, circumferential strain, and global three-dimensional strain were significantly decreased in the nondialysis patients compared with the other two groups (all, *P*<0.001). However, the three-dimensional strain and the regional longitudinal strain were lower in the hemodialysis patients than in the controls (*P*<0.01). In the hemodialysis patients and the control group, the longitudinal strain, circumferential strain, and radial strain were higher at the apical level than they were at the basal level and midlevels. A multivariate linear regression analysis showed that the blood urea nitrogen and creatinine levels were independently associated with the values of the global three-dimensional strain (β = −0.217, *P* = 0.000; β = −0.243, *P* = 0.011, respectively) and the longitudinal strain (β = −0.154, *P* = 0.032; β = −0.188, *P* = 0.029, respectively).

**Conclusions:**

Three-dimensional speckle-tracking echocardiography may detect myocardial dysfunction in patients with uremia who have preserved LVEF. The global three-dimensional strain and the regional longitudinal strain appear to be superior in hemodialysis patients compared with nondialysis patients.

## Introduction

Uremia is associated with an increased risk of cardiovascular diseases, including coronary artery disease, myocardial infarction, and heart failure. Mortality is increased by 10- to 20-fold in dialysis patients compared with healthy individuals [1]. Most studies have used the conventional echocardiographic parameters of cardiac function, such as ejection fraction and fraction shortening, which are frequently normal in uremic patients [2], [3]. A previous study showed that the ejection fraction and fraction shortening do not adequately describe the regional systolic function and the loading conditions affecting these parameters [4]. Attention has recently been focused on using Doppler tissue imaging to assess LV regional systolic function through the measurement of tissue velocities, displacement, strain, and strain rate. However, tissue Doppler imaging is limited by Doppler angle dependence, which can identify only the myocardial motion and deformation occurring in the direction of the ultrasound beam [5]. Therefore, it has been used primarily to assess longitudinal strain. However, longitudinal strain is only 1 of the 3 principle components of the regional systolic function [6]. Two-dimensional speckle-tracking echocardiography was also recently adopted to detect the subtle impairment of the LV systolic function in uremic patients who have normal LVEF [7], [8]. However, a major limitation of two-dimensional speckle-tracking echocardiography (2D STE) is that it is unable to quantify one of the three components of the local displacement vector because of the three-dimensional (3D) anatomy of the LV and the complex 3D patterns of the wall motion [9].

Three-dimensional speckle-tracking echocardiography (3D STE) is a new and emerging technique. It relies on 3D echocardiographic datasets and tracks the motion of speckles within a scanned volume, irrespective of direction. Yu et al. demonstrated that the 3D strain (3DS), which is best described as the “principle tangential strain,” may display the resulting direction's muscle fiber contractions, which are aligned either parallel to or tangential to the myocardial surface [10]. The combination of the three components, 3D optical flow and 3D phase correlation, as well as the 3D intensity correlation, allows the calculation of the 3D motion vector field of the subendocardium. Kleijn et al. confirmed the accuracy of 3D STE for the quantitative assessment of global LV function by using cardiac magnetic resonance imaging as a reference method [11], suggesting that 3D STE has the potential to overcome the limitations of Doppler tissue imaging and 2D STE [12], [13]. Therefore, the aim of this study was use 3D STE to investigate the LV global and regional myocardial function in hemodialysis and nondialysis uremic patients who have normal LVEF.

## Materials and Methods

### Study Participants

Between August 2012 and June 2013, 75 uremic patients with a LVEF of 55% or greater were consecutively enrolled from the nephrology clinic. The cases excluded from the study were 2 patients with arrhythmias, 2 patients with severe valvular disease, 3 patients with uncontrolled hypertension (i.e., systolic blood pressure greater than 140 mmHg and/or diastolic blood pressure greater than 90 mmHg), and 3 patients for whom the 3D STE image quality was inadequate. Finally, 65 patients with uremia were analyzed in this study. The uremia patients included 35 patients on maintenance hemodialysis (the hemodialysis group; 4-hour sessions, 3 times weekly for 3 months or more) who were prescribed a standard dose of dialysis targeting an equilibrated dose (Kt/V of urea) of 1.2 or more and 30 patients who were hospitalized for the creation of a primary arteriovenous fistula (the nondialysis group). We recruited 32 age-matched control volunteers from among individuals who received routine checkups at our hospital who had normal results on electrocardiography, transthoracic echocardiography, multi-slice spiral computed tomography (CT), and coronary angiography and had normal blood pressure. The biochemical indexes of all of the study participants were evaluated at the hospital laboratory. The indexes included total cholesterol, glyceride, hemoglobin, creatinine, and urea nitrogen levels in the blood. Written and informed consent was obtained from all of the study participants. The study was approved by the local ethics committee of the Sir Run Run Shaw Hospital (Hangzhou, China).

### Echocardiography Measurements

All of the echocardiographic studies were performed using a commercial scanner (iE33; Philips Medical System, N.A., Bothell, WA, USA). Real-time 3D echocardiographic imaging was performed from the cardiac apex by a fully sampled matrix-array transducer. The full-volume acquisition (which included 6 wedge-shaped volumes) was recorded with 6 consecutive cardiac cycles during a single breath-hold. Moreover, the temporal and spatial resolutions of the images were optimized by decreasing the depth and adjusting the sector width to ensure that the entire LV cavity was included within the pyramidal volume. An experienced echocardiographer obtained all of the echocardiographic images using a standard protocol, and another echocardiographer who was blinded to the clinical data of the study participants analyzed the images. An offline analysis of the data was performed using Tom-Tec 4.0 analysis software (Tom Tec Imaging Systems Gmbh, Unterschleissheim, Bayern, Germany). For the tracing of the endocardium and epicardium, the following five views were displayed: (1) the apical four-chamber view; (2) the apical two-chamber view and the three short-axis views; (3) the apex of the LV; (4) the midlevel of the LV; and (5) the basal level of the LV. The LV epicardium and endocardium were traced automatically, and the tracings were refined with further adjustment. Thus, in a cardiac cycle, the software was able to perform both global and segmental strain analyses, including the global 3D strain (3DS), regional longitudinal strain (LS), radial strain (RS), and circumferential strain (CS). Meanwhile, the end-systolic and end-diastolic LV volumes and ejection fractions were observed automatically. On the parasternal long-axis view, the thickness of the interventricular septum and LV posterior wall were measured at the end-diastole of the LV. The LV diameters were measured at the end-systole and end-diastole phases. The early (E) and late (A) diastolic peak velocities of the mitral inflow and the early (E′) and late (A′) diastolic peak velocities of the septal mitral annulus were measured by pulse wave Doppler and Doppler tissue imaging. Subsequently, the E/A ratio, E′/A′ ratio, and E/E′ ratio were calculated to evaluate the diastolic function of the LV.

### Intraobserver Reproducibility and Interobserver Reliability

For the intraobserver reproducibility, 10 subjects were analyzed twice within 4 weeks by one investigator, and the mean values of 16 segments at three levels of LV for RS, CS, LS and 3DS were calculated. The interobserver reliability assessment was performed by analyzing 10 subjects who were chosen randomly by 2 independent investigators. Both observers were blinded to the previous measurements.

## Statistical Analysis

All statistical analyses were performed using SPSS version 16.0 software (SPSS, Inc., Chicago, IL, USA). All data were presented as the mean ± the standard deviation (SD). The duration of uremia and hypertension were presented as median values. Differences in the continuous data and categorical data between the patients and the controls were compared using a one-way analysis of variance and χ^2^ tests. Independent determinations of subclinical myocardial systolic dysfunction in patients with uremia were examined using multivariate linear regression. The intraclass correlation coefficients (ICCs) method was used to assess the intraobserver reproducibility and the interobserver reliability for the values of the 3D global strain and regional strain. The ICCs were expressed as percentages. The clinical significance was categorized as follows: “excellent,” if the ICC was 0.80 or greater; “good,” if the ICC was between 0.61 and 0.79; “moderate,” if the ICC was between 0.41 and 0.60; and “poor,” if the ICC was 0.4 or less [14].

## Results

### Clinical Parameters

The general clinical indexes and the blood indexes of the hemodialysis group, the nondialysis group, and the control group are summarized in Table 1. Among the three groups, there were no significant differences in sex, age, heart rate, and blood pressure (all, *P*>0.05). However, the total cholesterol and glyceride levels were greater in the hemodialysis and nondialysis groups than in the control group (6.8±0.9 mmol/L and 8.0±1.2 mmol/L vs. 3.3±0.8 mmol/L; 4.8±1.2 mmol/L and 6.3±1.3 mmol/L vs. 1.2±0.8 mmol/L, respectively; *P*<0.01). The total cholesterol and glyceride levels were not significantly different between the hemodialysis and the nondialysis groups. The hemoglobin level was lower in the nondialysis group than in the hemodialysis group and the control group (8.5±0.6 g/dL vs. 11.3±0.8 g/dL and 8.5±0.6 g/dL vs. 12.4±0.9 g/dL, respectively; *P*<0.01). No difference was observed between the hemodialysis group and control group. The creatinine (CRE) and blood urea nitrogen (BUN) levels were significantly greater in the hemodialysis and nondialysis groups than in the control group (580.5±53.3 µmol/L and 897.8±186.9 umol/L vs. 100.5±26.3 umol/L; 20.6±6.4 mmol/L and 48.6±7.0 mmol/L vs. 6.8±1.3 mmol/L, respectively; *P*<0.001). These two levels were significantly decreased in the hemodialysis group compared with the nondialysis group (580.5±53.3 umol/L vs. 897.8±186.9 umol/L and 20.6±6.4 mmol/L vs. 48.6±7.0 mmol/L, respectively; *P*<0.001).

**Table 1:** Clinical Characteristics of the Uremia and Control Groups.

Parameters	Control (n = 32)	Hemodialysis (n = 35)	Nondialysis (n = 30)
Age (year)	53.05±6.83	52.78±7.65	53.16±5.69
Male/female	20/20	19/21	17/23
Course of disease (year)	--------	5.5±3.3	6.2±2.8
Heart rate (bpm)	75±11	73±10	74±12
Systolic BP (mmHg)	120±15	125±13	128±12
Diastolic BP (mmHg)	75±8	80±9	83±8
Body surface area (m^2^)	1.5±0.4	1.6 ±0.3	1.6±0.5
Total cholesterol (mmol/;L)	3.3±0.8	6.8±0.9*	8.0±1.2*
Glyceride (mmol/L)	1.2±0.8	4.8±1.2*	6.3±1.3*
Hb (g/dL)	12.4±0.9	11.3±0.8	8.5±0.6*†
CRE (umol/L)	100.5±26.3	580.5±53.3**	897.8±186.9** ††
BUN (mmol/L)	6.8±1.3	20.6±6.4**	48.6±7.0** ††
KT/V	NA	1.3±0.2	NA

BP = blood pressure; BUN  =  blood urea nitrogen; CRE  =  creatinine; Hb  =  hemoglobin; Kt/V  =  dose of dialysis.

*P*<0.01, compared with the control group.

**P<0.001, compared with the control group.

† *P*<0.01, compared with the hemodialysis patient group.

†† *P*<0.001, compared with the hemodialysis patient group.

### Echocardiographic Characteristics

The echocardiographic parameters of the study groups are shown in Table 2. Compared with the control group, both the nondialysis group and the hemodialysis group demonstrated higher left ventricular end-diastolic volume, left ventricular end-diastolic volume, left ventricular end-diastolic diameter, left ventricular end-systolic diameter, left ventricular septum thickness, left ventricular posterior wall thickness, and E/E′ ratio (all, *P*<0.01). However, there were no significant differences in these factors between the hemodialysis and nondialysis groups. The LVEF was similar in the three groups. Furthermore, the E, A, E/A ratio, E′, A′, and E′/A′ ratio (all, *P*<0.01) were decreased significantly only in the nondialysis group compared with the hemodialysis group and the control group. However, no difference was found between the hemodialysis group and the control group.

**Table 2:** Echocardiographic Characteristics of the Uremia and Control Groups.

	Control (n = 32)	Hemodialysis (n = 35)	Nondialysis (n = 30)
LVEDV(mL)	78.9±5.1	95.8±8.1*	109.4±7.2*
LVSDV(mL)	3 5.1±3.5	45.4±2.5*	54.3±2.9*
LVEDD(mm)	49.4±3.5	58.6±3.2*	60.2±2.8*
LVESD(mm)	30.5±4.3	41.3±2.6*	43.5±3.0*
LVEF(%)	62.9±2.4	57.8±1.9	56.7±2.0
IVST(mm)	9.2±0.4	13.8±0.5**	14.5±0.3**
LVPWT(mm)	8.8±0.3	12.8±0.2**	13.3±0.3**
E	85.3± 22.1	83.6±17.8	72.3±10.4*
A	78.61±2.5	85.3±20.1	93.9±19.4*
E/A	1.1±0.5	0.83±0.2	0.8±0.3*
E'	8.3±1.6	7.0±1.0	6.2±0.8*
A'	6.8±1.5	8.0±2.1	9.0±2.2*
E'/A'	1.12±0.3	0.92±0.2	0.71±0.1*
E/E'	10.3±2.7	14.3±2.5**	15.8±3.2**

A  =  transmitral peak velocity of late-diastole; E  =  transmitral peak velocity of early-diastole; A′, velocity of late diastole of the mitral annulus; E′  =  velocity of early diastole of the mitral annulus; E/A  =  the ratio of velocity of early and late diastole; E′/A′  =  ratio of velocity of early and late diastole of mitral annulus; IVST  =  interventricular septum thickness; LVEDV, left ventricular end-diastolic volume; LVEDD, left ventricular end-diastolic diameter; LVEF  =  left ventricular ejection fraction; LVESD, left ventricular end- systolic diameter; LVESV, left ventricular end-systolic volume; LVPWT  =  left ventricular posterior wall thickness.

*P<0.01, compared to the control group.

**P<0.001, compared to the control group.

### Speckle-Tracking Echocardiography Analysis

**Figure 1:**
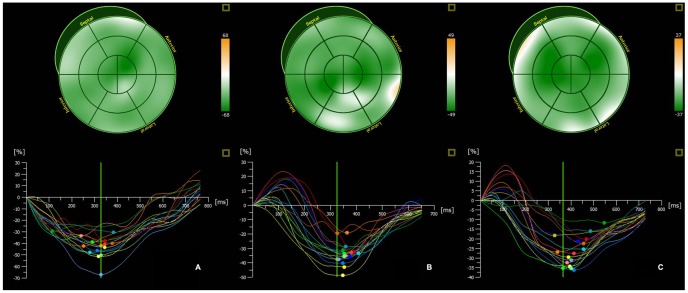
The three-dimensional strain curves of the 16 segments of LV in (A) the controls, (B) the patients in the hemodialysis group, and (C) the nondialysis patients. The color-coded curves indicate the different myocardial segments of the LV. The peak strain values of the different myocardial regions are more decreased in the uremic patients than in the controls. The peak strain is lowest in the nondialysis group.

One hundred and sixty (10%) of 1552 segments were excluded from the 3D STE analysis because of drop-out. The 3D STE analyses of the three groups are shown in Table 3. The LS, CS, and RS at the three levels of LV and 3DS were decreased in the nondialysis group compared with both the control group and the hemodialysis group (all, *P*<0.01) (Figure 1). In addition, the 3DS and LS at the three levels of LV were lower in the hemodialysis group than in the control group (all, *P*<0.01). In the CS and RS, there was no significant difference between the hemodialysis and the control group. The LS, CS, and RS were higher at the apical level than they were at the basal level and the midlevel in the hemodialysis patients and the control patients. However, these values were not significantly changed in the nondialysis group. Based on the multivariate linear regression analysis, the duration of the uremia, systolic blood pressure, LVST, E/E' ratio, and the levels of the total cholesterol, glyceride, Hb, CRE, and BUN were associated with the 3DS, LS, RS and CS, which were used to examine the risk factors for preclinical myocardial systolic dysfunction. The levels of CRE and BUN were independently associated with the 3DS value (β = −0.217, *P* = 0.000; β = −0.243, *P* = 0.011) and the LS value (β = −0.154, *P* = 0.032; β = −0.188, *P* = 0.029) (Table 4).

**Table 3:** 3D-STE parameters of the Uremia and Control Groups.

		Control (n = 29)	Hemodialysis (n = 30)	Nondialysis (n = 28)
Global	3DS(%)	−38.58±4.25	−25.52±6.81*	−17.75±5.87* ^,^ †
Basal	LS	−21.09±3.45	−16.98±2.35*	−10.78±3.08* ^,^ †
	CS	−40.63±2.96	−41.69±3.69	−28.58±5.10* ^,^ †
	RS	78.08±18.06	77.36±13.37	60.58±12.95* ^,^ †
Mid	LS	−22.96±2.65	−15.45±5.23*	−10.39±2.89* ^,^ †
	CS	−44.48±5.32	−42.39±4.56	−30.28±2.64* ^,^ †
	RS	92.37±8.69	90.78±6.31	64.58±5.87** ^,^ ††
Apex	LS	−32.43±4.51§ ^,^ ||	−23.87±3.41* ^,^ § ^,^ ||	−12.27±2.14* ^,^ †
	CS	−50.25±5.68§ ^,^ ||	−49.24±3.59§ ^,^ ||	−30.23±3.14** ^,^ ††
	RS	100.58±10.21§ ^,^ ||	101.58±16.31§ ^,^ ||	62.89±7.19** ^,^ ††

3DS  =  three-dimensional strain; CS  =  circumferential strain; LS  =  longitudinal strain; RS  =  radial strain; STE  =  speckle-tracking echocardiography.

*P<0.01, compared with the control group.

**P<0.001, compared with the control group.

† *P*<0.01, compared with the hemodialysis patient group.

†† *P*<0.001, compared with the hemodialysis patient group *P*<0.01, compared with the hemodialysis patient group.

§ *P*<0.01, compared with the values at the basal level.

|| *P*<0.01, compared with the values at the midlevel.

**Table 4:** Independent factors associated with the systolic functional parameters.

	3DS		LS		RS		CS	
	*β*	*P*	*β*	*P*	*β*	*P*	*β*	*P*
Duration of Uremia	0.074	.463	0.056	.379	0.021	.089	0.013	.089
Systolic BP	−0.125	.142	−0.074	.284	−0.058	.315	−0.252	.074
LVST	0.062	.326.	0.087	.365	0.021	.064	0.700	.125
E/E'	0.004	.245	0.025	.157	0.085	.310	0.018	.584
Total cholesterol	−0.035	.638	−0.042	.793	−0.038	.157	−0.098	.243
Glyceride	−0.041	.548	−0.058	.547	−0.024	.857	−0.035	.142
Hb	0.025	.336	0.102	.970	0.054	.634	0.047	.639
CRE	−0.243	.011	−0.188	.029	−0.116	.213	−0.137	.105
BUN	−0.217	.000	−0.154	.032	−0.128	.122	−0.092	.174

3DS  =  three-dimensional strain; CS  =  circumferential strain; LS  =  longitudinal strain; RS  =  radial strain; BP = blood pressure; IVST  =  interventricular septum thickness; E/E' = the ratio of transmitral peak velocity of early-diastole and velocity of early diastole of the mitral annulu; Hb  =  hemoglobin; BUN  =  blood urea nitrogen; CRE  =  creatinine; *β = Beta coeffident*

### Interobserver and Intraobserver Reproducibility

The ICCs are shown in Table 5. The ICCs were all above 0.8. The mean absolute percentage error of all of the measurements did not exceed 0.1. The intraobserver and interobserver reproducibility for the mean measurements of the LS, CS, and RS in the sixteen segments of the three levels of LV and 3DS were acceptable.

**Table 5:** Reproducibility of interobserver and intraobserver by 3D STE.

		Interobserver		Intraobserver	
		ICC	MAPE	ICC	MAPE
Global	3DS(%)	0.84	0.04	0.85	0.03
Basal	LS	0.85	0.05	0.81	0.05
	CS	0.87	0.07	0.91	0.08
	RS	0.85	0.05	0.89	0.07
Mid	LS	0.83	0.08	0.82	0.04
	CS	0.90	0.04	0.86	0.09
	RS	0.87	0.06	0.85	0.05
Apex	LS	0.81	0.08	0.87	0.07
	CS	0.84	0.07	0.90	0.06
	RS	0.82	0.06	0.84	0.05

3DS  =  three-dimensional strain; CS  =  circumferential strain; ICC  =  intraclass correlation coefficient; LS  =  longitudinal strain; MAPE  =  mean absolute percentage error; RS  =  radial strain.

## Discussion

Cardiac function is still incompletely characterized in uremia patients. This is the first study to compare the differences in the global and regional myocardial functions in hemodialysis and nondialysis patients based on 3D STE. The primary findings of this study are as follows: (1) the global LV systolic function (EF) was normal in the uremic group; however, the global 3DS and the regional LS, RS, and CS were decreased in the nondialysis group, indicating that the LV regional long-axis, short-axis, and global functions were already impaired; (2) the 3DS and LS were higher in the hemodialysis group than in the nondialysis group, indicating that the LV global and regional long-axis functions were superior in the hemodialysis patients; and (3) the decreased CS and RS were observed only in the nondialysis patients and not in the hemodialysis patients. These results demonstrate that, in myocardial dysfunction, the global LV dysfunction and LV regional long-axis systolic dysfunction appear to be more vulnerable to change compared with the short-axis function.

The LVEF reflects the sum of all of the regional shortening in the left ventricle, and regional wall motion impairment may not reduce the LVEF unless several segments are involved.

In our study, the LVEF was normal in the uremia group; however, the LV regional long-axis, short-axis and global functions were already impaired in the uremia patients as assessed by 3D STE, regardless of whether they were undergoing hemodialysis. The underlying mechanisms of the decreased LV function in uremia patients are complex and not thoroughly understood. We speculated that hypertension, LV hypertrophy, LV remodeling, coronary artery disease, myocardial fibrosis, and apoptosis may contribute to LV dysfunction in uremia patients with preserved LVEF. In addition, we found that the LV global function and regional long-axis function were superior in the hemodialysis patients, as shown by the higher 3DS and LS in the hemodialysis group than in the nondialysis group. We believe that the improvement in the LV function in the hemodialysis patients may have resulted from factors such as improvements in their anemia, malnutrition, calcium-phosphorus metabolism disorder and acid-base balance, etc. These factors may decrease the volume load of the LV and thereby improve the anoxic condition of the tissues and organs. The increase in the myocardial oxygen and blood flow as well as the improvement in the LV remodeling may also promote LV systolic function. Therefore, the results from the current study indicate that the 3DS and LS may be sensitive predictors of preclinical LV dysfunction in patients with uremia, particularly in hemodialysis patients. The detection of subclinical myocardial dysfunction may assist clinical doctors in treating uremia patients earlier (even if the LVEF is normal), and thereby reduce the incidence of cardiovascular disease while improving long-term outcomes.

Yan et al. also demonstrated that the CS and RS decreased only in the nondialysis patients based on the 2D STE [3]. This is similar to the results of our study, showing that the impairment in the short-axis function occurred later than the impairment in the regional long-axis function based on the 3D STE. The possible reasons for this result are (1) the augmented LV wall thickness and cross-fiber shortening, which exert their effects as the interaction between a contraction and a systolic increase in the cross-sectional area of all differently aligned myocardial layers; and (2) the curvature radius of the circumferentially oriented myocardial fibers is smaller than that of the longitudinal fibers, which may entail lower stress and consequently delay the appearance of functional abnormalities [15]–[17].

In addition, myocardial deformation occurs in three dimensions and can be characterized in the longitudinal direction, as well as in the circumferential and radial directions. Subepicardial fibers play a major role in both the RS and LS [18], and subendocardial fibers are important components in CS [19]. The subepicardium is more susceptible to myocardial ischemia and stiffness than the subendocardium. Maizel et al. reported that myocardial stiffness in the uremic mouse may represent an autonomic nervous dysfunction, cellular calcium, or an energy metabolism disorder, for example [20]. Our study showed that the CS and RS were observed in the nondialysis patients but not in the hemodialysis patients. Therefore, myocardial stiffness may be one factor that causes changes in the LV long-axis function earlier than in the short-axis function in uremia patients.

The LV's contractions travel from the apex to the basal region. Our study also found that the LS, CS, and RS were higher at the apical level than at the basal level and the midlevels in the control and hemodialysis groups. However, this phenomenon did not appear in the nondialysis group. A previous study has shown that myocardial contractility reflects the influence of preload [21]. We postulated that the improvement in water-sodium retention and the removal of fluid decrease the preload in hemodialysis patients compared with the nondialysis patients.

A decreased myocardial strain in uremia patients reportedly results from LV hypertrophy rather than from uremic toxicity [22]. In fact, in this study, the myocardial strain was superior in the hemodialysis patients, and the prevalence of hypertrophy was similar in the hemodialysis and nondialysis groups. Moreover, the multiple linear regression showed that the CRE and BUN levels were independent predictors of the global LV systolic function. The CRE and BUN levels were lower in the hemodialysis group than in the nondialysis group. We therefore assumed that the superior LV systolic function in the hemodialysis patients may result from the removal of uremic toxins. A previous study demonstrated that the injury of the myocardial cell membrane structure could cause cardiac dysfunction in uremic patients that was possibly related to the accumulation of uremic toxins [23]. Mark demonstrated that CRE could inhibit the activity of succinate dehydrogenase (SDH) in myocardial cells, which could lead both to obstacles in ATP production and mitochondrial swelling and fragmentation, and thus imbalance and damage to the nutrition and function of the myocardial cells [24]. Moreover, in some studies, BUN is a powerful predictor of increased mortality in patients hospitalized for heart failure. In older patients hospitalized for heart failure [25], a positive linear relationship was found between the BUN and the occurrence of 1-year mortality. According to our study, both the BUN and the CRE possibly contributed to myocardial dysfunction to a certain degree. In addition, indoxyl sulfate (IS), a uremic toxin, can induce TGF-βand phospho-NFkB p65 protein expression, and the reactive oxygen species-NF-kB-TGF-β_1_ pathway may play a role in cardiac fibrosis. Lekawanvijit et al. showed that cardiac fibrosis is reversible by reducing the serum IS level in uremic rats [26]. Therefore, the reduction in uremic toxins possibly alleviates the injury to the myocardial function in hemodialysis patients.

## Study Limitations

The temporal and spatial resolutions are relatively lower in the 3D STE than in the 2D STE. Therefore, several patients had to be excluded because the image quality in 1 or more segments was insufficient for the STE analysis. Moreover, our study included a relatively small number of patients. In addition, the long-term cardiovascular event rates or survival assessment in the patients on maintenance hemodialysis was not presented in this study.

## Conclusions

Three-dimensional STE analysis offers a useful tool for predicting the myocardial function of the LV in uremic patients. The global LV systolic function and the short-axis and long-axis regional LV myocardial function may be impaired in nondialysis patients who have preserved ejection fraction. However, the global LV function and regional LV systolic function appeared to be superior in the hemodialysis patients over those in the nondialysis patients. 3DS and LS may be sensitive predictors of LV global and regional systolic dysfunction.
